# The Delay of Old Age and the Alleviation of Senility1The oration on medicine at the fifty-sixth annual meeting of the American Medical Association, held at Portland, Ore., July 11-14, 1905.

**Published:** 1905-08

**Authors:** Charles G. Stockton

**Affiliations:** Buffalo, N. Y., Professor of Medicine in the University of Buffalo; 436 Franklin Street


### BUFFALO MEDICAL JOURNAL.

VOL. LXI. AUGUST, 1905. NO. 1

### SPECIAL ARTICLE.

### The Delay of Old Age and the Alleviation of Senility.[1]

BY CHARLES G. STOCKTON, M. D., Buffalo, N. Y.,
Professor of Medicine in the University of Buffalo.

IN 1805, Lewis and Clark, typical and celebrated among American explorers, turned their faces toward the farthest west and disclosed in a single expedition the possibilities of the continent and the mysteries of the great undiscovered. Their feat was in principle but a repetition of the undertakings of Columbus, De Soto, and La Salle,—of Daniel Boone and the numberless pioneers who so quickly, almost suddenly, transformed America from a desolate wilderness into the puissant commonwealth of today. In each we may discern a discontent with the established order and environment of his heritage; an inspiration to find in newness a satisfaction which the settled regions, settled habits of thought, settled manners of life failed to provide him at home. The word "settled" is significant. Our race is without rest until it settles things; and with this there stalks a poignant impatience with the things that are settled. The new, the undiscovered, the unattained, the unprecedented,—this it is which charms American manhood. The muezzin calling his prayers from a minaret in far away Cairo, the yellow haze in the circumambient air, the camels with jingling bells, the palms turned always eastward by the hot breeze from the monotonous sands,—these possess for the western man no attraction greater than that of poetic interest. In these scenes he could never make his life. The European finds in America everywhere that which impresses him with our youth;

1. The oration on medicine at the fifty-sixth annual meeting of the American Medical Association, held at Portland, Ore., July 11-14, 1905.

in the Orient, he sees the indisputable evidence of age and a settled order of things. This exploitation of new lands, the cry of "Westward Ho!" which for the past few centuries has stirred mankind, is rapidly, inevitably dying out; but during the long period of its continuance it has wrought important changes in our point of view, it has stimulated a luxuriant growth of thought, it has modified considerably our nature. As our common interest has been fixed upon the untried, we have gradually come to exaggerate the importance of new truths and to ignore the meritorious which the slow moving caravan of thought has brought us from the ages.

### MIDDLE AND LATE AGE HAVE USEFULNESS.

Now, it may seem a *non sequitur,* but reflection leads one to inquire if the great appreciation which we feel for the vigorous youth-spirit of recent times, has not educated us to prize,—perhaps to overvalue,—youth to the disparagement, if not to the absolute neglect, of old age.

In saying this we are not unmindful of the element of truth in the right interpreted utterance on this subject of one of our most distinguished colleagues. From the standpoint of the original investigator, as from that of the soldier, or the adventurer, there is nothing to replace the effort of the man under forty; but if for no other reason than to obstruct impetuosity and to provide such a handicap that youth shall not too soon outrun the limitations which a wise Providence has provided, middle and late old age have their uses. At any rate the old man is with us and there appears to be no acceptable method of obliterating him, even if it would add to the welfare of the race to do so.

Finding ourselves in this obvious position, it would seem the wise course so to manage that the disheartening effects of years may be as long as possible postponed; and to seek those methods by whose application we may do our utmost to minimise the terrors of senility. If youth is so delectable, let us find how it may be protracted; if old age is so beset with ineptitude, let us not rest until we learn how it may be mitigated. Such a proposal is not quite identical with a scheme for the prolongation of life—mere increase in years, although it would doubtless include this, however, desirable, or regrettable it might be. It has to do directly with something about whose desirability there can be no doubt. In other words, I ask your attention to the question of the practicability of *keeping* the various functions of the organism, of preserving the physical and intellectual powers, of delaying the onset of degeneration as much as may be, of developing a race less prone to early decay.

Then, besides this, I wish you to consider the feasibility of so managing the senile when once their degeneration has arrived, that many of their miseries may be avoided and the remainder lessened in degree. Having granted that age is inevitable the average man would seem to have become indifferent to his penalty for living, and, if complainingly, he none the less heedlessly allows his forces to ebb away prematurely. With some the whole of existence seems to be spent in infancy, adolescence, and senility: There is no period in which one can say, "there is full, healthy maturity." When examined this early senility is found to result from pathologic processes and there the observation ends as though it were conclusive. But the event is not thus to be disposed of, for, according to our best standards, senile changes are always pathologic, and, therefore, the question of prematurity rests on some pathologic reason for cause, or else upon inherent weakness in human vitality. We are in the habit of speaking and thinking of senility as something separate and apart from disease; as a result, not sufficient attention has been given to the question of prevention of age.

THE THEORY OF METCHNIKOFF.

On this subject one cannot escape the bold work and bolder thought of Metchnikoff, and I quote at once his description of the degenerations of age: "In senile atrophy the same condition is always present: the atrophy of the higher and specific cells of the tissue and their replacement by hypertrophied connective tissue." It is this same process on which every teacher of medicine constantly harps in discussing disease, the disappearance of the parenchyma and the increase of the stroma.

One need not dwell on the theory of Metchnikoff as to the rôle of the phagocytes in the process; for the moment it is enough to know and recognise that senile atrophy is pathologic and that, essentially, old age is disease. It is unnecessary to discuss the plausible but startling suggestion—that of finding some antiserum which might antagonise the development of the age-producing agencies. Already we possess some knowledge of how to defer age, but this information is not generally insisted on as a guide in living. We frequently allude to measures of prevention, but we do not reduce them to a system which can be taught to the student and to society. If we are further to lengthen the probational study period in medicine, it will be wise to give the matter of deferred old age very careful consideration. If the subject were studied seriously, we would foresee a tremendous improvement in the type of humanity, and the readjustment of many habits, customs, social questions along altogether new lines.

### A LACK OF IMPORTANT DATA.

Obviously, real progress cannot be made without accummulating data from which to deduce positive conclusions. At present we lack information on such simple matters as the effect on the organism of diet, exercise, recreation and rest. There are many opinions, but they are largely theoretical. To begin with, we must know something about the material with which we have to deal; that is, the inheritance, the reserve powers, the internal resistance of the individual. In fact, we know very little concerning the inheritance of any one. All are more or less obscure in the matter of family history. There are courts of records in which are found descriptions of estates, but there are no archives in which are deposited accurate descriptions of the various members of the family itself.

We are more particular in relation to our domestic animals, especially in the introduction of a new strain. Inquiries are made as to the matter of endurance, longevity, powers of nutrition, good or bad qualities of mind or body, but concerning ourselves, there is an indifference to these important qualities of organic perfection. It would not seem to be altogether impossible to acquire some official record of the physical and intellectual qualities; in other words, strains in men. It is not improbable that if such records were known to exist, they would be not only consulted, but would gradually grow to have an influence on the formation of families.

### IMPORTANCE OF CONTROL OF MARRIAGE CONTRACTS.

Love is said to know no barriers, but history fails to confirm this view. If a matter of class, of caste, or rank, has been sufficiently important to control the forming of marriage contracts, it is not impossible to conceive of a more intelligent public sentiment which would make an alliance with a family celebrated for physical, intellectual and moral superiority, a thing greatly to be desired; and conversely, which would lead to the abhorrence of an alliance with a family in which freaks, degenerates and incompetents, or in which a tendency toward lowered nutrition, too early maturity, or premature senility were frequent events.

Naturally this is not an affair of a generation, but it is unquestionably a doctrine which ought to be taught. The notion that acquired characteristics can have any transmitted influence on the character of the offspring, as formerly taught by the Lamarckins, is still doing great harm. Our young people should be instructed in the fact that the well being of their posterity depends almost altogether on the native stamina of the germplasm with which they are endowed. They should be taught to understand the responsibility implied in maternity and paternity, and should be made to recognise that the greatest influence which they can have on the well-being of their children is in the selection of the spouse. The sentiment should be encouraged that parentage is a matter which concerns the public, and especially posterity, more than it does the parent; that the germ-plasm of the race is of all questions the most important which concerns it. Measures could be adopted which would make it more possible than at present for individuals to inform themselves concerning the family characteristics of the contemplated fiancée. A bureau for the precise registration of disease and causes of death, together with the chief facts in family and personal history, might be established through legal enactments, reinforced and made active by a high social sentiment. Such a bureau would afford the needed information, and the special study of the material thus provided would result in the formation of guiding principles in human biology and race development.

### DEVOLUTION NOT TO CONTINUE.

Such a system might seem to be contrary to our laws of personal rights, and, of course, the sanctity of reserve may not be easily disrupted. Nevertheless, with Emerson, "We shall one day see that the most private is the most public energy; that quality atones for quantity," and although we cannot attain at a bound to these heights of wisdom, we may, at least, begin to map out a way. He is short-sighted who supposes that the complacency shown in the devolution of the race must continue, unchanged, eternally. We have only admiring applause for the remarkable accomplishment of Burbank in the vegetable world, and yet such are our deep-rooted prejudices that we shrink from the application of similar principles to human uplifting even when we know it is possible. Shaler remarks that man "sorely needs a herdsman's care." The subject is painful, yet I think it will be acknowledged that susceptibility toward tuberculosis, insanity, alcoholism, epilepsy, and the like, is hereditary. Granting this, it follows that a stock could be developed which practically would be immune to those diseases. Doubtless all will regard the realisation of this suggestion to be remote and some may liken it to that of Maeterlinck, who says: "Man may sometime master the secret of gravitation and by means of it steer his planet wherever in the universe he wills." The principle is open to such wide application that it may easily be obscured in absurdity. Nevertheless, here is an honest straightforward truth not so far beyond our reach. It lies in a public demand that none but healthy parents shall be allowed to bear children. The brevity of middle age resides, first, in the inherited quality of the organised tissue; second, in the effects of its environal conditions on each organism. We physicians are making it our function to exterminate disease. Why should we be content with merely pointing out that certain disease tendencies are hereditary without taking some practical steps to prevent such disease. Why should we be content with merely pointing out that certain disease tendencies are hereditary without taking some practical steps to prevent such disease transmission? As an illustration, it doubtless would be a hardship to the syphilitic to make procreation for him a penal offense. Nevertheless, such a course would be full of beneficence to the race. The amount of nervous and other disease in the innocent resulting from syphilis in progenitors is incalculable. The incompetence and misery which often overtake the unfortunate victim in middle life as the result of syphilis, one or two generations removed, must be considered. Those who have studied the subject carefully, regard this disease as responsible for one-fifth of all cases of arteriosclerosis, and of yet a higher proportion in those cases occurring in middle life. A man has not the moral right to beget disease. Notwithstanding that it usually comes through ignorance, it should not be allowed. The profession of medicine should make its convictions bear fruit, and should teach and should be expected to teach that the marriage of a large number of people whose nuptials they now help to celebrate should either be prohibited or else made barren. To those who would regard the realisation of these thoughts as unattainable, it may be remarked that the world is yet very young, and as a result, as Carlisle has said, in most things very stupid. At any rate it will be admitted that middle age could be considerably prolonged and the infirmities of old age largely mitigated if we could eliminate from the equation that faulty cell metabolism which arrives through inheritance.

### THE DEVELOPMENT OF THE INDIVIDUAL.

The trend of modern thought is toward the development of the individual. Our researches in therapeutics, dietetics, immunity, gymnastics all alike are directed toward the betterment of the individual by making life possible to many who would otherwise perish, comfortable to many who would otherwise survive in misery,—result in prolonging life. Our educational efforts concern themselves more and more with fitting for life the otherwise unfit, in so dealing with the neurotic, the semi-imbecile, the vicious and criminal that they may be practical units in the body politic. The result is that individuals otherwise impossible are so modified that they are finding some happiness in life and become less disturbing elements in society as a whole. There can be no doubt as to the benefit to the individual, and no doubt as to the good which comes from the reflex effect on society. Perhaps nothing counts more for the moral uplifting of the masses than the awakening of personal interest in all altruistic undertakings, but let me repeat, we must dismiss as untenable the views of the Neo-Lamarckians that acquired characteristics of the individual have any appreciable effect on the offspring. It is acknowledged that the natural tendency of defectives of all kinds is toward extermination. Left to themselves, many of these weak strains would disappear. In making it possible for these strains to continue to blend and to dilute the organic type of the race, we are unquestionably lowering the future standard of internal resistance and we are creating a threat to physical, intellectual and moral perfection.

On this subject Professor Woods Hutchinson of this city (Portland) has said some interesting things.

Is it unchristian or unethical to consider this question from the phylogenic standpoint, or, on the contrary, is it not from the highest ethical plane that we look forward to a means that may elevate man spiritually, strengthen him intellectually, increase his vital and physical vigor, thus widening the period of his usefulness and decreasing his sufferings as a whole? We find ourselves sustained in our efforts when they are directed toward helping the individual, but there seems to be a social veto placed on the study of generic betterment. Weismann and his followers have shown us that the character of the individual depends on the germ-plasm with which he starts life, and although education and favorable environments may modify greatly the individual, they have no effect whatever in improving the character of his posterity. The possibilities of an individual, his resistance to disease, his longevity, his strength and his intelligence are determined beforehand by his inheritance. It is known that it is possible to bring deterioration by introducing an inferior strain.

### EDUCATION CANNOT COUNTERACT INBREEDING OF BAD STOCK.

No educational advancement can counteract the injurious effects of this preservation and inbreeding of the bad stock. Unless intelligent foresight be used to prevent this definite and ascertained source of degeneracy, we are likely to find a growing depreciation of our racial stock and, in spite of our modern preventive medicine, a progressive increase of mental and physical disease, and an abbreviation of the working years. By homely methods we have learned that aside from the question of perfect nutrition and proper exercise of function, there is nothing which contributes so unquestionably toward the improvement of organic life as intelligent study of what is known as breeding, in other words by the intelligent blending of the germ-plasm of healthy, resisting, long-lived stock so that there may result not inferior progeny, but offspring superior to the parental stock. I am aware that the question is not new, and that its mere mention exposes one to criticism, not only from the timid and ignorant, but even from the wise and philanthropic, and yet, no one can deny that in shutting our eyes to this truth which Nature spreads plainly before us and which she makes the subject of countless lessons illustrated by individual inferiority, perversity, disease and death, we are not doing all that we ought for the coming race, not all that we might toward assisting that evolution which we hope for, but which lingers so in the process. It will be said that no one can contemplate such a scheme as is here suggested without feeling strongly convinced of its impracticability. Of course, it must be admitted that the large fulfilment of such ideals is at present quite impracticable, but it is not impracticable to study the subject with the hope of finding certain aspects which may admit of modification.

### INDIVIDUALISM SHOULD RECOMPENSE SOCIETY.

By a study of the subject we would at least offer a reasonable antidote to the possible injurious effects of the modern rage for individualism. More than this, it may be possible, by more or less united action, so to modify established customs, social tendencies and common law that we may elevate the sense of family, and possibly municipal and national morality to a standard high enough to result in our placing the welfare of the community above the desires and the instinct of the individual. As we have somewhat meddlesomely diverted the natural tendency of deterioration and degeneration from its end, thus interfering with the action of the law of the survival of the fittest, it does not seem improper that individualism should recompense society by yielding some of its prerogatives for the welfare of the race; or, to put it more pointedly, it would seem only fair, since we have preserved the life of the weakling, that we should deny him the privilege of paternity, or since we have educated the defective or criminal youth into semi-useful citizenship that we should forbid him the right of increasing the number of his kind. In one of our states a legislative enactment looking toward this end was recently vetoed by the governor, who accompanied his decision by a conventional lecture to the profession of medicine on its willingness to disregard the rights of individuals. This illustrates one of the difficulties in the way, and would seem to indicate still another field in which physicians must assume their rôle of public educators. The sentiment of the people is instinctively against public interference with personal privilege. This feeling seems to be particularly strong in the Anglo-Saxon people, and to be contrary to the nature of our institutions; yet, like every other privilege it is susceptible of doing great evil if carried to its full logical extreme.

Are we not as an organic type suffering from an extreme application of individual rights? Are we not inflicting an injury on the race through permitting unwholesome privilege to the individual? Is it necessarily dangerous to attempt to create a public sentiment, a social feeling toward the making of new customs and the enactment of new laws which may result in the elimination of at least some of the more abhorrent and destructive elements that at present are blending with and antagonising the evolution of the race? Would it not be desirable to have an aristocracy of health? That is to say, to see developed a zeal for physical and intellectual perfection, and to have that zeal supported by public sentiment, so that there might be created something like a caste, the attributes of which should be mental and physical wholesomeness, and from which should be excluded those families which are shown by carefully studied data to come of degenerate or unfit stock? This would result in a contest between the general and the particular. At present the particular, the individual, prevails; in the presence of rightly adapted social feeling the general would prevail. Evolution would thereby be speeded and not retarded as at present. The principle involved does not seem to be any more unethical than the application of the quarantine laws which now operate injuriously to the individual, but make greatly for the public good.

### HOW WE MAY POSTPONE AGE.

Let us now consider what may be accomplished in the way of postponing age by the reasonable treatment of even the poor material that comes to us. Undoubtedly the question is as broad as the whole subject of personal hygiene and general therapeutics, and so, for the most part, must be passed; there are, however, some aspects of the subject which deserve special consideration. One of these is the improvement in the nutrition of the aged as the result of good teeth. While all recognise the comfort and convenience which we owe to the dentists, it is doubtful if we fully appreciate how much they have contributed to good health and longevity. For another great advance we have to thank the oculists. Who can estimate the additional resources both of usefulness and happiness secured through the discovery of spectacles and the operation for cataract? Useful eyesight contributes much toward health and long life for the reason that it permits of a continued interest in living which otherwise would be lost. It permits of a struggle for an ideal and in this real happiness consists. The ideal, of course, is never reached, but, as Montaigne says, the reward is not found in the victory, but in the conflict. Perhaps no one factor is so important in maintaining courage and health in old people as the creation and continuance of some keen interest in life, and this interest, even in the otherwise enfeebled, may be kept up by means of useful eyes. Now comes the time worn but neglected subject of arterial disease.

Too often our attention is for the first time attracted to arteriosclerosis by the appearance of fibroid or fatty degeneration, exhibiting itself in the figure, form and viscera of the individual. Even after these later changes appear measures may be adopted to stay the march of the process, but how much better it would be to anticipate it. For instance, by noting the high and rising blood pressure and the physical signs of cardiac strain, by estimating the decline in urinary solids and the rise in the urotoxic unit, by measuring the decline in respiratory capacity, or by detecting the falling off in cutaneous secretion and the loss of the pliability and softness of the skin, we may make ourselves aware that vascular degeneration is progressing. Heedless of these earlier manifestations we shall soon awake to the advent of the state which Sir James Paget has so marvelously described.

In the earlier steps of arteriosclerosis, if intelligent study be given to the individual, to his habits of life, to his excesses and to his deficiencies, very much can be done, and, by thoughtful men, is done, to delay arterial changes and to restore functional activity.

May I trespass so far as to emphasise the importance of judging and correcting the disturbed balance between assimilation and waste—the anabolic and katabolic processes? The facts are so well understood that one hesitates to mention them, and yet just here there is so much neglect of precious opportunity. There are successful methods of lessening the extent of autointoxication and of widening the field for the play of nutritional processes. This is so commonplace that we neglect it. Middle age often brings luxury and at the same period the contracting arteries narrow the field of physiologic activities. The circumstances of life create taste in the man for indulgence in appetite or for excess in undertakings, while the limitations of the organism are increased. The tendencies are thus in opposite directions, and, with Munsterberg we ask, "How can they keep together who are going different ways?" Ultimately, they must separate, of course, but temporarily they may keep company if we help to preserve harmony between them. There is abundant proof that even advanced arteriosclerosis may be so far modified as to delay the oncoming degeneration and to admit of happy usefulness, but the victim must be content with moderation, and not hope like Mahomet to cleave the moon in twain.

Advanced arteriosclerosis is too generally neglected, and intoxications of one kind or another are for the most part responsible for it. Our prophylactic and curative measures are readily understood. It is well known that the relatively great fatality of pneumonia and other acute diseases is explained by the greater toxemia which exists late in life. Besides this, the autointoxication increases the blood pressure and induces prematurely fatal events of cerebral, cardiac and renal diseases. Almost as deplorable as these are the conditions of unnatural excitement, confusion and depression from which many people unnecessarily suffer in old age. Still further, there may be found in vascular disease the source of much severe pain from which old people suffer. This pain is often referable to the aorta, at times to those forms of aortitis and periaortitis of the abdomen recently so well set forth by Teissier, of Lyons. The latter has also shown that the pain can be ameliorated, the progress of the disease stayed and even great improvement induced by rational treatment. Aortitis and periaortitis often greatly interfere with the flow of blood into the branches of the abdominal aorta, and as a result the organs supplied by these branches suffer special degeneration. In middle age and later life, intractable diseases of the stomach, kidneys and other organs could be dealt with much more successfully, and comfortable longevity secured, if more attention were devoted to this pathology.

### HOW TO MAKE OLD AGE MORE TOLERABLE.

There remains for brief consideration the question of what can be done to make old age more tolerable; in other words, to remove it to a physiologic basis. We find on close consideration that most of the derangements from which the aged suffer can be classified as belonging distinctly to pathology, but I am afraid there exists a tendency among us to dismiss these matters as necessary corollaries of senility, without giving them that careful consideration which similar processes receive in younger patients. To a few the diseases of old age have a peculiar charm, and these few who give senile diseases special study seem to agree that the complaints of the aged arise for the most part from toxic causes.

Even the threadbare question of arteriosclerosis falls to a large extent under this head. There is good reason for believing that this toxic state which underlies the decadence of senility takes its origin for the most part in the colon.

Metchnikoff supports the view that mammals have developed a large colon for the purpose of storing the products of digestion, and that man has inherited a large colon at the expense of his longevity. This organ harbors an immense number of bacteria, leading to fermentations, putrefactions and the production of alkaloids, fatty acids and toxins which man has to combat for the length of his mortal days. While vitality is young and vigorous, the combat is not so unequal, but with failing powers autointoxication prevails in the contest between the internal resisting powers of the organism and the toxic adversary made potent by inheritance. Unfortunately the degeneration of age first produces functional insufficiency of the organs of elimination, then their degeneration, and as a result we find the skin, kidneys, lungs, liver, incompetent in each individual overtaken by years. It is somewhat remarkable that facts so well understood as these do not receive more attention.

There are few textbooks, there is little teaching devoted to the pathology and right management of senile disease, and yet experience teaches that few patients respond more satisfactorily than do the aged if their cases are approached with the principles in mind which are here set forth. It is not merely a question of dilatation of the colon, nor of any special defect of the organ; even in the absence of these, the source of autointoxication exists and becomes important because of the failure of the skin and other emunctories which, earlier in life, are relatively sufficient. The indications are obvious. In addition to the usual measures for improving the general circulation, old people are benefited by systematic colonic lavage, stimulating baths with superficial massage, prescribed pulmonary gymnastics and an abundant drinking of pure water. It is the almost universal effect of these matters which has made it seem to me a subject of sufficient importance to call to your attention in a general address. There is something inspiring about youth which gives an incentive to our care of the diseases of childhood and adolescence, but there is something benignant in studying the problems that lead to the postponement of age and to the alleviation of the trials of senility. Old age is repulsive when it is pathologic, but it is beautiful when it is physiologic, and it would appear as a fitting theme for the consideration of this great body at its meeting in this new land of the setting sun.

436 Franklin Street.

